# Impact of COVID-19 Awareness on Protective Behaviors during the Off-Peak Period: Sex Differences among Chinese Undergraduates

**DOI:** 10.3390/ijerph192013483

**Published:** 2022-10-18

**Authors:** Teng Zhao

**Affiliations:** Zhejiang Academy of Higher Education, Hangzhou Dianzi University, Hangzhou 310018, China; zhaoteng@hdu.edu.cn

**Keywords:** COVID-19 awareness, undergraduate students, protective behaviors, structural equation modeling, sex difference, China

## Abstract

COVID-19 remains an extreme threat in higher education settings, even during the off-peak period. Appropriate protective measures have been suggested to prevent the spread of COVID-19 in a large population context. Undergraduate students represent a highly vulnerable fraction of the population, so their COVID-19 protective behaviors play critical roles in enabling successful pandemic prevention. Hence, this study aims to understand what and how individual factors contribute to undergraduate students’ protective behaviors. After building multigroup structural equation models using data acquired from the survey taken by 991 undergraduates at a large research university in eastern China, I found that students’ COVID-19 awareness was positively associated with their protective behaviors, such as wearing a mask, using hand sanitizer, and maintaining proper social distance, but not with getting vaccinated. In addition, I found students with higher COVID-19 awareness were more likely to have more COVID-19 knowledge than those with less awareness. Furthermore, sex differences were observed in the mediation effects of COVID-19 awareness on wearing a mask and getting vaccinated, via COVID-19 knowledge, respectively. The results of this study have implications in helping higher education stakeholders enact effective measures to prevent the spread of the pandemic.

## 1. Introduction

Though many countries across the world have begun to cope with COVID-19, discrimination against infected individuals in society is still on-going [1]. Particularly in the labor market, employers are likely to discriminate against employees who have been previously infected but have recovered because of the “better safe than sorry” proverb [2]. Other than claiming equal rights, adopting appropriate and effective behaviors to prevent the infection is a plausible alternative [3]. As the most populated country in the world, China has endured tremendous pressure from the rest of the world to curb the pandemic, especially in its higher education system, as college students are highly vulnerable to disease transmission because of their clustering. Thus, it is crucial to understand how undergraduate students, the largest component of the college student population, take protective measures to prevent the spread of the disease.

Prior research has indicated that the dissemination of detailed communication of pandemic-related information from governments to the general population significantly contributes to the public’s protective behaviors, such as wearing a mask and improving hand hygiene [4]. During emergency situations and crisis management, risk communication has been found to increase risk awareness among the public [5]. Furthermore, disseminating disaster/crisis information is intended to enrich the public’s knowledge to correctly respond to disasters/crises [6]. As such, undergraduate students’ protective behaviors to prevent the disease may be affected by how aware they are of the pandemic and how knowledgeable they are. This is consistent with a study by Li et al. [7], who found that American participants’ risk awareness of COVID-19 was significantly associated with their engagement in preventative behaviors. In addition, Ning et al. [8] highlighted that Chinese citizens with higher knowledge were more likely to have higher levels of protective behaviors than those with less knowledge.

Given the periodic recurrence of the COVID-19 pandemic, Chinese governments have adopted a new anti-epidemic policy that aims to adjust the severity of control measures based on the severity of the pandemic [9], and these anti-epidemic policies at academic institutions are in sync with the policies implemented by the governments. Generally, undergraduate students take sufficient protective measures when pandemic outbreaks occur, not only because of the students’ self-willingness but also due to the mandatory requirements of academic institutions. The past literature has found that COVID-19 awareness and knowledge contributed to protective behaviors during the peak of the COVID-19 outbreak in China [8,10,11]. However, it is unclear how much the mandatory requirements contributed to these protective measures. Therefore, off-peak periods provide a unique condition to assess whether, how, and to what degree undergraduate students’ awareness and knowledge about the COVID-19 pandemic affect their protective behaviors.

The present study seeks to answer these questions. In addition, it also attempts to understand whether and how these effects vary by sex. This study contributes to the existing COVID-19 protective behavior literature by focusing on the off-peak period of COVID-19 and the large but vulnerable population of undergraduate students. To my knowledge, the current literature fails to address both what and how questions (i.e., what factors affect protective behaviors and how) and to examine simultaneously the sex differences among these behaviors and relationships. Using a sample of Chinese undergraduate students, I collected data during an off-peak COVID-19 period by conducting a literature-based, self-designed survey and used that data to build multigroup structural equation models (SEM). The purpose of the research study was to serve the research purposes and discuss the important practical implications for higher education stakeholders based on the findings.

## 2. The Present Study and Rationale

**Figure 1:**
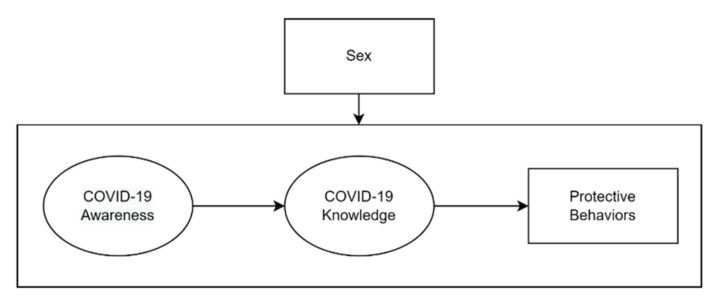
The hypothesized model of this study.

Multigroup SEM is an appropriate approach for the present study not only because it can reliably examine the hypothesized relationships among intended variables, but it also helps to understand how these relationships vary by sex. The hypothesized model developed in this study is shown in Figure 1. According to Dai et al. [4], the public’s risk perception can significantly predict their protective behaviors. Differently, this study adds a mediation factor—knowledge of COVID-19—to help understand the “how” question. My rationale to add this mediation factor was that undergraduate students who perceive COVID-19 as a higher risk (i.e., awareness) ought to proactively seek COVID-19 information, such as how the disease spreads and how to prevent it (i.e., knowledge). In turn, with adequate knowledge of COVID-19, undergraduate students ought to understand what types of protective behaviors could be adopted.

To continue, as indicated by a variety of the existing literature [7,12,13], the most common protective behaviors to prevent being infected with COVID-19 include wearing a mask, thoroughly washing hands, and maintaining adequate social distance. These three protective measures, in addition to getting vaccinated against SARS-CoV-2, represent the four protective behaviors that were examined in the model. Additionally, past literature has documented that there were differences in COVID-19 awareness, knowledge, and protective behaviors between the sexes [12,14,15,16]. Thus, this study also examined the sex differences in the intended variables as well as the relationships/paths in the tested model.

## 3. Materials and Methods

### 3.1. Data and Sample

The data used to build the models in this study were collected from a self-designed survey: College Students’ Epidemic Preparedness (CSEP). The survey items were designed based on those from Ahmed et al. [17] and Ikhlaq et al. [18], which mainly focused on students’ COVID-19 awareness, knowledge, and protective behaviors. In addition, the survey collected students’ individual information, such as sex, ethnicity, grade, and college major, as well as their familial information, such as annual household income and parental education.

The survey was distributed to undergraduate students in a large research university located in eastern China via an online survey platform called WJX.CN. The period of survey collection was from May to June 2021, when the entire country was under the “Normalized Epidemic Prevention and Control Requirements” during the off-peak period of the pandemic [19]. The participants were informed that their personal information could not be identified through the survey. After validating the survey, only five invalid responses were found; these included surveys with either missing information or that contained incorrect information. Due to the low percentage of missing values, I adopted a listwise deletion method that yielded a final analytic sample of 991, which included 680 (68.62%) male students and 311 (31.38%) female students. With respect to ethnicity, 940 (94.85%) of the students were Han—the socially dominant group in China—and 51 (5.15%) of the students were of the other 55 minority ethnicities, such as Miao and Zhuang. In terms of grade, 393 (39.66%) were freshmen, 281 (28.36%) were sophomores, 160 (16.15%) were juniors, and 157 (15.84%) were seniors.

### 3.2. Measures

#### 3.2.1. Endogenous Variables

The students’ knowledge of COVID-19 and protective behaviors were considered as endogenous variables in this study. The knowledge of COVID-19 was measured by two survey questions: “I know how COVID-19 spreads” and “I know how to protect from COVID-19”. The responses were assessed based on the five-point Likert scale, with 1 = strongly disagree and 5 = strongly agree. The students’ protective behaviors included four actions: wearing a mask, using hand sanitizer, maintaining proper social distance, and getting vaccinated. Each of these action items was solely measured by a single question. Among them, the responses to the first three questions were assessed using a five-point Likert scale, while getting vaccinated was assessed using a three-point scale (see detailed descriptions of the variables in Table 1).

#### 3.2.2. Exogenous Variables

As the only exogenous variable in the present study, the students’ COVID-19 awareness was measured by three survey items. Undergraduate students were asked to rate “the frequency of talking about COVID-19 to classmates”, “the frequency of talking about COVID-19 to family members”, and “the frequency of talking about COVID-19 to friends”. The responses ranged from “never” to “always”, with 1 = never and 5 = always (see detailed descriptions of the variables in Table 1).

### 3.3. Analytic Process

The analysis of the present study was conducted by *Mplus* 8. Since this study also aimed to understand whether there were sex differences in these hypothesized relationships/paths, I firstly reported the differences in the descriptive statistics between women and men. Second, because all of the intended survey items were categorical, polychoric correlation analysis was conducted to provide a statistical rationale to measure the latent variables (i.e., COVID-19 awareness and knowledge). Third, to build the multigroup SEM, all the parameters in the initial model were freely estimated in both sex groups; this model was considered as the unconstrained model. To decide which equality constraints were selected, I added equality constraints of paths and covariances one by one to the unconstrained model and conducted chi-square difference tests in sequence [20], starting from the comparison between the unconstrained model and a slightly more constrained one. A diagonally weighted least squares estimator with mean and variance adjusted (WLSMV) was then applied due to the polychoric correlations [21].

After identifying the equality constraints, I assessed the constrained model to see if it was adequate based on the model fit indices, such as the comparative fit index (CFI), the Tucker–Lewis fit index (TLI), the root mean square error of approximation (RMSEA), and the standardized root mean square residual (SRMR). In addition, I compared it to the initial model to determine which model was better, and the better model was considered the final multigroup structural model. Lastly, to understand whether the students’ knowledge of COVID-19 played a mediation role on the effects of the students’ COVID-19 awareness of protective behaviors, four mediation models (Awareness to Knowledge to Wearing mask, Awareness to Knowledge to Using sanitizer, Awareness to Knowledge to Maintain social distance, Awareness to Knowledge to Getting vaccinated) derived from the final multigroup SEM model were assessed using Sobel’s test [22].

## 4. Results

### 4.1. Descriptive Statistics and Polychoric Correlation Results

Table 2 displays the descriptive statistics and polychoric correlations of the variables in this study. For the male students in this study, the means of Items 1–3 that measured awareness were 2.329, 2.241, and 2.207, respectively, while the same items were 2.328, 2.264, and 2.241, respectively, for the female students. When assessing the knowledge of COVID-19, the means of Items 4 and 5 in the male group were 3.131 and 3.200, respectively, which were both slightly higher than the respective values (2.974 and 3.125) in the female group. Looking at the students’ protective behaviors, male students scored 2.110, 2.212, 2.191, and 2.868 for the items regarding wearing a mask, using hand sanitizer, maintaining proper social distance, and getting vaccinated, respectively, while women scored 2.141, 2.264, 2.232, and 2.859 in the corresponding behaviors.

Effect size analysis was performed to preliminarily check whether the differences in these variables between the male and female students were statistically significant. The results showed that the male students scored significantly higher with regard to knowledge of COVID-19 than the female students, with Cohen’s *d* = −0.163, 95% CI (−0.297, −0.028). However, no significant differences in the other variables between the two groups were observed.

With respect to the polychoric correlations, Table 2 also shows that the correlations among Items 1–3 ranged from 0.805 to 0.908 for the male students and from 0.814 to 0.888 for the female students, while Items 4 and 5 were 0.876 for the male students and 0.850 for the female students. These relatively high correlations suggested that the latent factors (i.e., awareness and knowledge of COVID-19) could be adequately measured by these survey items.

### 4.2. Selection of the Final Multigroup Structural Model

To determine whether or not certain parameters could be equality constraints, I performed chi-square difference tests among the different models, and the results are shown in Table 3. When only constraining the equality in the covariance between wearing a mask and maintaining social distance between the female and male students, the model was significantly different, with *χ*^2^(1) = 8.549, *p* < 0.01. Then, I added this equality constraint into the initial unconstrained model, as the constrained model. The constrained model yielded a good model fit in terms of Hu and Bentler [23], with CFI = 0.999, TLI = 0.998, RMSEA = 0.029, SRMR = 0.018, and *χ*^2^(49) = 69.099. Since no modification indices were suggested by *Mplus,* I did not further assess a modified constrained model.

Yet, considering whether the constrained model was better than the unconstrained model, a chi-square difference test was conducted between these two models. The result showed statistically significant differences between the two models, with Δ*χ*^2^ = 8.508, Δ*df =* 1, *p* < 0.01, indicating that the more complicated model (the unconstrained model) was preferred. Furthermore, I assessed the model fit of the unconstrained model and obtained even better model fit indices, with CFI = 0.999, TLI = 0.999, RMSEA = 0.023, SRMR = 0.017, and *χ*^2^(48) = 60.591. Therefore, the initial (unconstrained) model was selected as the final multigroup structural model.

### 4.3. Final Multigroup Structural Model Results

Prior to reporting the results of the paths, the factor loadings of the latent variables (i.e., awareness and knowledge) were presented. The standardized factor loadings for the female students’ COVID-19 awareness and knowledge ranged from 0.846 to 1.005, while the range was from 0.854 to 1.018 for male students, suggesting these survey items measured the latent variables well. For example, talking about COVID-19 to family in the female group had a factor loading of 0.938, indicating that 87.98% of the variance in “talking about COVID-19 to family” was explained by COVID-19 awareness.

**Figure 2:**
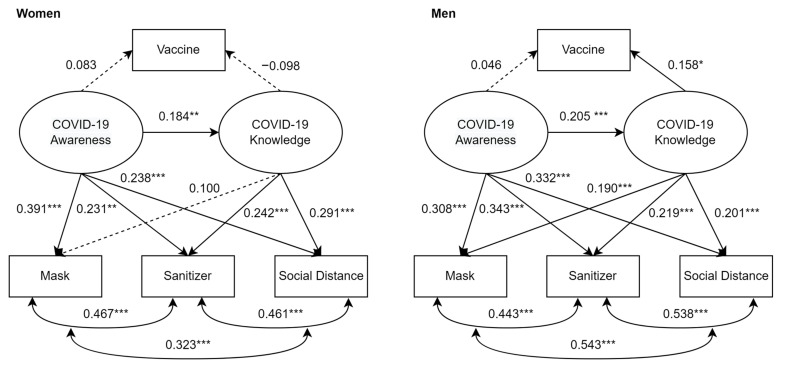
The final multigroup SEM model (unstandardized). Note: * *p* < 0.05, ** *p* < 0.01, *** *p* < 0.001. Statistically insignificant results are presented as dash arrows.

Figure 2 displays the paths/relationships and covariances among the variables. Results showed that both the female and male students’ COVID-19 awareness was positively associated with their knowledge (women: *b* = 0.184, *p* < 0.01; men: *b* = 0.205, *p* < 0.001). In addition, both female and male students’ COVID-19 awareness positively predicted some of their protective behaviors, such as wearing a mask (women: *b* = 0.391, *p* < 0.001; men: *b* = 0.308, *p* < 0.001), using hand sanitizer (women: *b* = 0.231, *p* < 0.01; men: *b* = 0.343, *p* < 0.001), and maintaining social distance (women: *b* = 0.238, *p* < 0.001; men: *b* = 0.332, *p* < 0.001), but not getting vaccinated (women: *b* = 0.083, *p* > 0.05; men: *b* = 0.046, *p* > 0.05).

Furthermore, in both the female and male groups, the students’ knowledge of COVID-19 significantly contributed to using hand sanitizer (women: *b* = 0.242, *p* < 0.001; men: *b* = 0.219, *p* < 0.001) and maintaining social distance (women: *b* = 0.291, *p* < 0.001; men: *b* = 0.201, *p* < 0.001). However, statistically significant and positive relationships between COVID-19 knowledge and wearing a mask (*b* = 0.190, *p* < 0.001) and getting vaccinated (*b* = 0.158, *p* < 0.05) were found in the male students, but not in the female students.

### 4.4. Mediation Effects of COVID-19 Awareness on Protective Behaviors

Since COVID-19 awareness significantly predicted that the students would wear a mask, use hand sanitizer, and maintain social distance, corresponding mediation effects were tested via COVID-19 knowledge, respectively. Using the *indirect* command in *Mplus* and conducting Sobel’s test [22], Table 4 presents that significant and partial mediational relationships in the paths to wearing a mask (z = 3.309, *p* < 0.001), using hand sanitizer (z = 3.354, *p* < 0.001), and maintaining social distance (z = 3.482, *p* < 0.001) were observed in the male student group. The results also showed partially mediating relationships in the paths to using hand sanitizer (z = 2.461, *p* < 0.05) and maintaining social distance (z = 2.650, *p* < 0.01) in the female student group. However, no mediational effects were observed in the path to the female students’ wearing a mask. Notably, a full mediation effect was observed in the path from COVID-19 awareness to getting vaccinated in the male student group (z = 1.983, *p* < 0.05).

## 5. Discussion

It is novel to examine the relationships among undergraduate students’ awareness and protective behaviors by developing a hypothesized model, and to jointly assess the mediational role of the knowledge of COVID-19 from the sex perspective in the context of the off-peak pandemic period. The present study also aimed to understand the sex differences in the undergraduates’ COVID-19 awareness, knowledge, and protective behaviors as well as the relationships/paths among these variables. There were three major findings from the study. First, the male students scored higher on the knowledge of COVID-19 than the female students. As to awareness and protective behaviors, no significant sex differences were found. Second, both female and male students’ awareness significantly predicted all four protective behaviors (i.e., wearing a mask, using hand sanitizer, maintaining social distance, and getting vaccinated). Third, both male and female students were found to support the mediation of knowledge of COVID-19 in the paths to using hand sanitizer and maintaining social distance, but different mediation effects to wearing a mask and getting vaccinated were observed.

Male students were more knowledgeable about COVID-19 than their female peers. This finding was consistent with that of a previous study conducted in China with a larger undergraduate student sample including 13 universities and colleges [14]. In Bangladesh, Hossain et al. [24] found that men aged from 13 to 88 scored higher on knowledge of COVID-19 than women. Using a sample of 6639 students from 22 countries, Riad et al. [25] also found a similar result, which indicated that men had higher levels of perceived knowledge sufficiency. It is plausible that different sex groups have different preferences of categories of knowledge, which could be partially explained by sex stereotypes [26]. For example, men are typically more interested in the traditional masculine fields, such as chemistry and biology, than women, which might be the reason why men are more interested in acquiring knowledge of COVID-19. In addition, Klein [27] found that women were more likely to avoid news that induce anxiety and fear. It is likely that female students avoid COVID-19 news, preventing them from obtaining as much information/knowledge about COVID-19 as males.

In this study, both male and female students’ COVID-19 awareness contributed to their protective behaviors: wearing a mask, using hand sanitizer, maintaining social distance, and getting vaccinated. This may not be a surprise because many countries’ gov-ernments have attempted to use TV and social media to increase public awareness and urge their citizens to adopt protective behaviors [16,28]. After surveying 5761 Chinese participants across the country, Sun et al. [29] reported that approximately 100% of the public was aware of COVID-19, and nearly 100% of the public was compliant with wearing a mask. Focusing on using hand sanitizer, Rundle et al. [30] pointed out that the COVID-19 pandemic increased the awareness of hand cleansing across the world. It is reasonable to conclude that individuals with higher awareness of COVID-19 are more likely to improve hand hygiene, such as using hand sanitizer, which is consistent with my findings.

With respect to social distancing, my result was consistent with Qazi et al. [31], who found that the situational awareness of participants in Brunei Darussalam led to the adoption of social distancing. In China, Xie et al. [32] surveyed 317 residents and found that their risk perceptions had positive effects on social distancing. Notably, the null finding of the relationship between awareness and getting vaccinated was likely due to other factors such as governmental vaccination enforcement. Specifically, those students with less awareness of COVID-19 risk who have lower intentions to get vaccinated, in fact, might still be required to get vaccinated.

It is worthy to note that this study also found that both male and female students have partial mediational effects in the paths to using hand sanitizer and maintaining social distance, but different mediational effects in the paths to wearing a mask and getting vaccinated. Different from Iorfa et al. [33], which set COVID-19 risk perception as the mediator between knowledge and precautionary behavior, this study treated knowledge as the mediator between awareness and protective behavior. The variation in the selection of the mediator was largely due to the policy differences between countries. China has adopted strict anti-epidemic policies since the beginning of the outbreak, which may enable the Chinese public to more likely perceive the risk of COVID-19 prior to acquiring knowledge of COVID-19. This contributed to the research gap in the mediational effects of knowledge between COVID-19 awareness on protective behaviors.

The results of mediational effects further supported that knowing what COVID-19 is and how to prevent it both play a crucial role in linking COVID-19 awareness to properly using hand sanitizer and maintaining social distance in both male and female students. Interestingly, no mediational effects of knowledge were observed in the path from awareness to getting vaccinated in female students, but a fully mediational effect was observed with the male students. This was probably attributed to women being more likely to be sensitive to different types of vaccine, making them assess the importance of the COVID-19 vaccine in their overall health. For example, women are likely to place a priority on the HPV vaccine rather than the COVID-19 vaccine, especially because it is not recommended that people receive the COVID-19 vaccine at the same time as other vaccines. Therefore, women may pay less attention to learning about COVID-19 than men. In addition, some studies have found women are more likely to perceive risks than men [34]. This might cause women to avoid receiving the COVID-19 vaccine since it was just developed during the survey time. This might also help to explain why I observed sex differences in the mediational effects of knowledge between awareness to wearing a mask; the reason why women wear masks was largely due to their cautiousness rather than knowledge. The mediating effect observed in the present study suggested that informing students, particularly females, more about COVID-19 could help them cope with COVID-19 prevention and thereby avoid being infected.

Three limitations of this study should be noted. First, although the survey of this study was comprehensively designed, a lot of information was still not collected. For example, more survey items of protective behaviors, such as staying in residential locations [35], could be collected and examined along with the other protective measures. This could add new insights to practical implications about how to effectively adopt protective behaviors against COVID-19. Second, other factors such as individual and family characteristics [36] may also affect students’ protective behaviors. These variables were not included in the study, since the main purpose of the present study was to mainly investigate the relationships among awareness, knowledge, and protective behaviors. Future research could adopt the propensity score matching technique to control these observable variables and draw causal inferences from the data. Third, though I used multiple survey items to measure latent variables, other relevant survey items also could be added. For example, for students’ awareness of COVID-19, how frequently a student searched COVID-19 information online could also measure a student’s awareness. This was not collected by the present study, which could be furtherly explored by future studies. However, the results of this study are still valuable for higher education administrators to make informed decisions to increase students’ awareness and knowledge, thereby making students better understand protective behaviors against COVID-19.

## 6. Conclusions

Preventing students from being infected with SARS-CoV-2 is a strategy that is central to the daily operations of higher education systems. This study sought to understand not only the effects of COVID-19 awareness on protective behaviors and to examine how knowledge of COVID-19 can mediate the effects of awareness on protective behaviors but also how sex plays a role in these effects. The results indicate that students’ awareness was significant and positive in predicting their wearing masks, using hand sanitizer, and maintaining social distance, but not in getting vaccinated. Notably, sex differences were observed not only in the knowledge of COVID-19 but also in the mediation effects of awareness on wearing a mask and getting vaccinated. These findings are helpful to inform higher education administrators how to help students adopt effective protective behaviors against COVID-19 during off-peak periods.

## Figures and Tables

**Table 1:** Description of variables.

Variables	Variable Description	Variable Type
Exogenous Variables		
COVID-19 awareness		
Talking about COVID-19 to classmates	The frequency of talking about COVID-19 to classmates (5-point Likert scale, with 1 = never and 5 = always)	Categorical variable
Talking about COVID-19 to family	The frequency of talking about COVID-19 to family members (5-point Likert scale, with 1 = never and 5 = always)	Categorical variable
Talking about COVID-19 to friends	The frequency of talking about COVID-19 to friends (5-point Likert scale, with 1 = never and 5 = always)	Categorical variable
Grouping Variable		
Sex	Whether a student is male or female (1 = male, 0 = female)	Dichotomous variable
Endogenous Variables		
COVID-19 Knowledge		
Knowledge of spread	Knowing how COVID-19 spreads (5-point Likert scale, with 1 = strongly disagree and 5 = strongly agree)	Categorical variable
Knowledge of protection	Knowing how to protect from COVID-19 (5-point Likert scale, with 1 = strongly disagree and 5 = strongly agree)	Categorical variable
Students’ protective behaviors		
Wearing a mask	The frequency of wearing a mask (5-point Likert scale, with 1 = never and 5 = always)	Categorical variable
Using hand sanitizer	The frequency of using hand sanitizer (5-point Likert scale, with 1 = never and 5 = always)	Categorical variable
Maintaining social distance	The frequency of maintaining social distance (5-point Likert scale, with 1 = never and 5 = always)	Categorical variable
Getting vaccinated	Willingness to get COVID-10 vaccine (3-point Likert scale, with 1 = disagree, 2 = neutral, 3 = agree)	Categorical variable

**Table 2:** Descriptive statistics and polychoric correlations of intended categorical variables, by sex.

												Male (*N* = 680)	
Variables	Min	Max	1	2	3	4	5	6	7	8	9	Mean	SD
1. Talking about COVID-19 to classmates	1	5	-	0.805	0.908	0.183	0.208	0.321	0.338	0.326	0.074	2.329	0.805
2. Talking about COVID-19 to family	1	5	0.833	-	0.811	0.157	0.196	0.312	0.373	0.333	0.019	2.241	0.785
3. Talking about COVID-19 to friends	1	5	0.814	0.888	-	0.134	0.177	0.326	0.362	0.372	0.112	2.207	0.763
4. Knowledge of spread	1	5	0.131	0.144	0.110	-	0.876	0.233	0.233	0.237	0.163	3.131	0.982
5. Knowledge of protection	1	5	0.169	0.173	0.199	0.850	-	0.243	0.305	0.267	0.155	3.200	0.944
6. Wearing a mask	1	5	0.330	0.284	0.368	0.112	0.168	-	0.540	0.615	0.070	2.110	0.941
7. Using hand sanitizer	1	5	0.186	0.250	0.234	0.190	0.271	0.572	-	0.629	0.047	2.212	0.990
8. Maintaining social distance	1	5	0.141	0.256	0.312	0.258	0.278	0.437	0.572	-	0.044	2.191	0.920
9. Getting vaccinated	1	3	0.153	-0.057	0.009	-0.073	-0.053	-0.154	-0.110	-0.056	-	2.868	0.406
	Female	Mean	2.328	2.264	2.241	2.974	3.125	2.141	2.264	2.232	2.859		
	(*N* = 311)	SD	0.737	0.673	0.669	0.919	0.884	0.774	0.930	0.818	0.424		

Note: Correlation between two ordinal variables was reported by polychoric correlation. SD = standard deviation. The results of the female group were presented in the lower triangle, and the male group’s were presented in the upper triangle.

**Table 3:** Chi-square difference tests for choosing equality constraints for multigroup SEM.

Paths/Covariances	*χ*^2^(1)	*p* Value
Paths		
Awareness to Knowledge	0.010	0.920
Knowledge to Wearing a mask	1.431	0.232
Awareness to Wearing a mask	0.617	0.432
Knowledge to Using hand sanitizer	0.008	0.928
Awareness to Using hand sanitizer	2.287	0.130
Knowledge to Maintaining social distance	0.936	0.333
Awareness to Maintaining social distance	1.090	0.297
Knowledge to Getting vaccinated	3.467	0.063
Awareness to Getting vaccinated	0.135	0.713
Covariances		
Wearing a mask with Using hand sanitizer	2.246	0.134
Wearing a mask with Maintaining social distance	8.549 **	0.004
Maintaining social distance with Using hand sanitizer	0.067	0.796

Note: ** *p* < 0.01. *N* = 991.

**Table 4:** Mediation effects of COVID-19 awareness on students’ protective behaviors (unstandardized).

Paths	Women (*N* = 311)				Men (*N* = 680)			
	Direct	Indirect	Total	Sobel	Direct	Indirect	Total	Sobel
Awareness → Knowledge → Wear mask	0.391 ***	0.018	0.409 ***	1.242	0.305 ***	0.039 ***	0.344 ***	3.309 ***
Awareness → Knowledge → Use sanitizer	0.231 **	0.045 *	0.275 ***	2.461 *	0.340 ***	0.045 ***	0.385 ***	3.543 ***
Awareness → Knowledge → Maintain SD	0.238 ***	0.053 **	0.292 ***	2.650 **	0.329 ***	0.041 ***	0.369 ***	3.482 ***
Awareness → Knowledge → Get vaccinated	0.083	−0.018	0.065	−0.788	0.046	0.032 *	0.078	1.983 *

Note: * *p* < 0.05, ** *p* < 0.01, *** *p* < 0.001.

## Data Availability

Data are available upon reasonable request.
